# Cesarean Scar Ectopic Pregnancy: A Retrospective Study of Ultrasound-Guided Suction Evacuation From Saudi Arabia

**DOI:** 10.7759/cureus.78883

**Published:** 2025-02-12

**Authors:** Nouar M Elzewawi, Amina Salhi, Hafasa Khalid, Shaden AlMojel, Ammar Mallisho, Mamoun M Elawad

**Affiliations:** 1 Department of Obstetrics and Gynaecology, King Abdullah Bin Abdulaziz University Hospital, Riyadh, SAU; 2 Department of Obstetrics and Gynaecology, King Salman Hospital, Riyadh, SAU; 3 Department of Obstetrics and Gynaecology, Ministry of Health, First Health Cluster, Riyadh, SAU; 4 Department of Radiology, King Abdullah Bin Abdulaziz University Hospital, Riyadh, SAU

**Keywords:** cesarean scar ectopic pregnancy, csep, saudi arabia, suction evacuation, β-human chorionic gonadotropin

## Abstract

Objectives

Globally, as rates of cesarean section increase, so do the rates of cesarean scar ectopic pregnancies. The objective of this study is to describe our experiences in managing cesarean scar ectopic pregnancy (CSEP) in King Abdullah Bin Abdulaziz University Hospital.

Method

This retrospective cohort study was conducted at King Abdullah Bin Abdulaziz University Hospital, Riyadh, Saudi Arabia. The study included women of reproductive age who presented with a cesarean scar ectopic pregnancy (CSEP) and a diagnosis confirmed by the transvaginal ultrasound. The suction evacuation was performed by ultrasound-guided suction evacuation. All the relevant data was retrieved from medical records.

Results

We reported ten CSEPs managed by ultrasound-guided suction evacuation. All ten patients successfully underwent ultrasound-guided suction evacuation for CSEP without complications. Most of the patients experienced minimal blood loss, except one case developed excessive blood loss, which was controlled by balloon tamponade. All the patients were dischargeable on the same day of the procedure or the following day, and they experienced a significant decrease in beta-human chorionic gonadotropin (HCG) levels, ultimately reaching undetectable levels, apart from one case in which the histopathology confirmed partial molar pregnancy and is still undergoing follow-up by beta-HCG till the date of study publication. All patients were discharged with advice for an early scan in subsequent pregnancies. The study revealed ultrasound-guided suction evacuation's effectiveness, safety, and advantages as a minimally invasive and reasonable approach to managing early CSEP.

Conclusion

Ultrasound-guided suction evacuation can be safely and effectively applied to CSEP management, and it remains one of the most cost-effective methods.

## Introduction

Cesarean scar ectopic pregnancy (CSEP) is rare; with increasing cesarean section rates, more cases are encountered, especially in busy maternity hospitals. It is associated with severe complications such as uterine rupture, bleeding, morbidly adherent placenta, and maternal death [1]. Appropriate, timely diagnosis is better to avoid these complications. The increased number of cesarean sections performed globally has led to a rise in CSEP cases, which pose significant challenges in terms of diagnosis and management [2,3].

However, more management schemes, including medical and surgical treatment options, have been reported in the last few years [4-7]. Diagnosing CSEP usually involves a combination of clinical history, transvaginal ultrasound, and sometimes magnetic resonance imaging (MRI) to determine the extent of CSEP and confirm the diagnosis [8]. Several management options have been advocated for the CSEP [9,10]. In some studies, surgical management is superior to medical management [11,12]. However, such surgical procedures are often accompanied by lengthy recovery periods, high costs, and associated risks of complications [13]. Various management modalities have been tried with different results [14-16].

By communicating our management experiences and the rationale for some of our recommendations, we hope to participate in the debate on managing such a challenging clinical problem. This article described the management of early-diagnosed scar ectopic pregnancy by suction evacuation (10 weeks and less), which was guided by ultrasound, and described our experiences in managing cesarean scar ectopic pregnancy at King Abdullah Bin Abdulaziz University Hospital.

## Materials and methods

Study design

The study was a retrospective cohort study conducted at King Abdullah Bin Abdulaziz University Hospital in Riyadh, Saudi Arabia. It includes women of reproductive age who presented with suspected cesarean scar ectopic pregnancy (CSEP), confirmed through transvaginal ultrasound and, in some cases, MRI. The success rate of the procedure was determined by the complete resolution of the ectopic pregnancy, with potential complications identified and reported. Considering the nature of the study, primary data collected included relatives’ demographics, clinical presentation, ultrasound and MRI results, management plans, histopathology, and follow-up data. The collaboration lasted from October 2022 to June 2024 and entailed a large-scale demographic profile and assessment of any complications during prognosis. The application’s effectiveness was seen by establishing the total resolution of the ectopic pregnancy, a testament to the potential positive outcomes of the procedure. At the same time, possible complications were recorded as well.

Participants

Participants in the study were women of reproductive age who presented with a suspected cesarean scar ectopic pregnancy (CSEP). The diagnosis was confirmed through transvaginal ultrasound and, in some cases, by MRI. Data for the study was obtained and managed from the electronic record system, ensuring accurate and comprehensive information collection for analysis. 

Inclusion and exclusion criteria

The study included all women with a confirmed diagnosis of cesarean scar ectopic pregnancy (CSEP) based on ultrasound, with a gestational age of fewer than 12 weeks, and who had provided informed consent for participation. However, women with hemodynamic instability and those with contraindications to suction evacuation were excluded from the study.

Data collection

The data collection process was done for the demographic and clinical information. This included ultrasound and MRI examinations to verify the diagnosis of CSEP and determine the appropriate intervention. We recorded the chosen management approach and the histopathological findings during ultrasound-guided suction evacuation. Post-treatment data were collected to follow up on the decrease in beta-human chorionic gonadotrophin (hCG) levels and the overall efficacy of the intervention.

Doppler study

Doppler ultrasound can identify increased vascularity around the gestational sac, which is often seen in ectopic pregnancies, including CSEP. This information is vital for clinicians to make informed decisions regarding the management and surgical approach, ensuring patient safety and minimizing the risk of excessive bleeding.

Surgical procedure details

The surgical procedure involved ultrasound-guided suction evacuation performed under general anesthesia and aseptic conditions to ensure safety and accuracy. Preoperative precautions included obtaining informed consent and ensuring the patient was hemodynamically stable. The suction evacuation procedure was meticulously carried out under continuous ultrasound guidance to ensure the complete removal of the ectopic tissue while minimizing damage to the surrounding myometrium. Blood loss was estimated intraoperatively, with most cases reporting minimal blood loss, except for one case where excessive bleeding was controlled using balloon tamponade.

Blood loss estimation

Blood loss estimation was performed by measuring the volume of blood collected during the procedure and visually assessing the surgical field. This method allowed for accurate monitoring of blood loss and ensured timely intervention if excessive bleeding occurred.

Follow-up

As per hospital protocol, the patients were constantly monitored for follow-up or to assess the improvement of their symptoms and the decline of beta-hCG levels. This indicates thorough elimination of the ectopic tissue and further potential complications that are ready for management at any time. This comprehensive follow-up approach ensured that patients received continuous care and support, contributing to the overall effectiveness and safety of the treatment method.

Data analysis

The characteristics of the participants were determined using descriptive statistics, including their clinical presentations, relevant findings, and treatment outcomes. The success rate of ultrasound-guided suction evacuation was calculated as the proportion of patients with complete resolution of ectopic pregnancy. Potential complications were also identified and reported. 

Ethical concerns

The Institutional Review Board of our institute waived ethical approval for the study. As the study was conducted in a teaching hospital, all patients provided written informed consent for using their data upon admission. The electronic record system of the hospital review, as the retrospective study, ensured that ethics in terms of trust, transparency, and, most importantly, integrity of the study and the respondents were protected and maintained throughout the whole study process, which defines ethical standards of the research about humans.

## Results

All ten patients successfully underwent ultrasound-guided suction evacuation for CSEP without complications. Most of the patients experienced minimal blood loss, except one case developed excessive blood loss, which was controlled by balloon tamponade. All the patients were dischargeable on the same day of the procedure or the following day, and they experienced a significant decrease in beta-HCG levels, ultimately reaching undetectable levels, apart from one case in which the histopathology confirmed partial molar pregnancy and is still undergoing follow-up by beta-HCG till the date of study publication. All patients were discharged with advice for an early scan in subsequent pregnancies. The outcomes reflected the effectiveness, safety, and advantages of power ultrasound-assisted suction evacuation as a minimally invasive and reasonable approach for treating early CSEP. 

Table 1 shows the key features of the ten cases in the study managed by ultrasound-guided suction evacuation. The table highlights the patient characteristics. The cohort studied consisted of women in their mid-thirties to early forties and with three to five prior cesarean sections. Most of the women were around six to nine weeks of gestation when CSEP was diagnosed. The clinical presentation varied among the patients; while some experienced mild lower abdominal pain, others had minimal vaginal bleeding, and others were asymptomatic.

**Table 1:** The key information from the cohort cases (n=10) GA: gestational age; hCG: human chorionic gonadotropin

Case	Age	GA	Previous Cesarean Sections	Symptoms	Beta-HCG (mIU/mL)	Procedure	Blood Loss	Outcome
1	36	7	5	Mild lower abdominal pain	83,468	Ultrasound-guided suction evacuation	100ml	Complete resolution
2	34	7	4	Mild lower abdominal pain	51,007	Diagnostic hysteroscopy and suction evacuation	100ml	Complete resolution
3	41	6	4	Asymptomatic	129,190	Ultrasound-guided suction evacuation	50ml	Complete resolution
4	43	7	3	Brownish discharge	27,721	Ultrasound-guided suction evacuation	20ml	Complete resolution
5	40	8	2	Minimal vaginal bleeding	86,811	Ultrasound-guided suction evacuation	30ml	Complete resolution
6	44	7	5	Brownish vaginal discharge	7,232	Ultrasound-guided suction evacuation	20ml	Complete resolution
7	34	9	3	Asymptomatic	185,466	Ultrasound-guided suction evacuation	500ml	Complete resolution
8	44	7	4	Minimal vaginal bleeding and lower abdominal pain	9,355	Ultrasound-guided suction evacuation	50ml	Complete resolution
9	32	8	2	Lower abdominal pain	126,993	Ultrasound-guided suction evacuation	50ml	Undergoing follow up (partial molar pregnancy)
10	38	10	2	Vaginal bleeding	15,329	Ultrasound-guided suction evacuation	150ml	Complete resolution

All patients exhibited significantly elevated beta-hCG levels, confirming the presence of a pregnancy. Ultrasound-guided suction evacuation was successful in all cases, demonstrating the safety and effectiveness of this approach.

Case 1 

**Figure 1:**
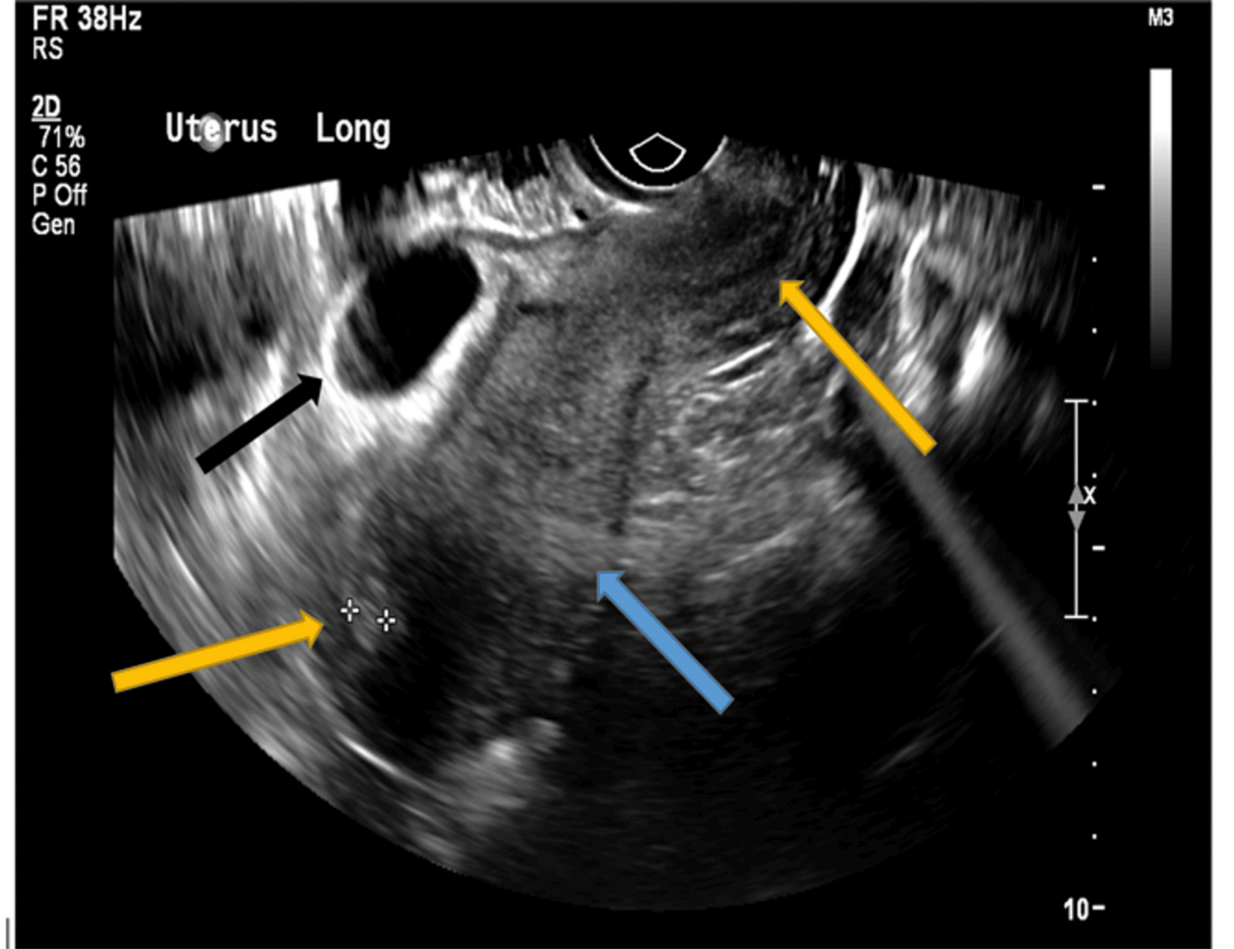
Longitudinal transvaginal ultrasound showing an ectopic cesarean scar pregnancy The black arrow indicates the thinning of the anterior aspect of the myometrium. The blue arrow indicates the normal thickness of the posterior myometrial wall. The yellow arrows indicate empty endometrium.

**Figure 2:**
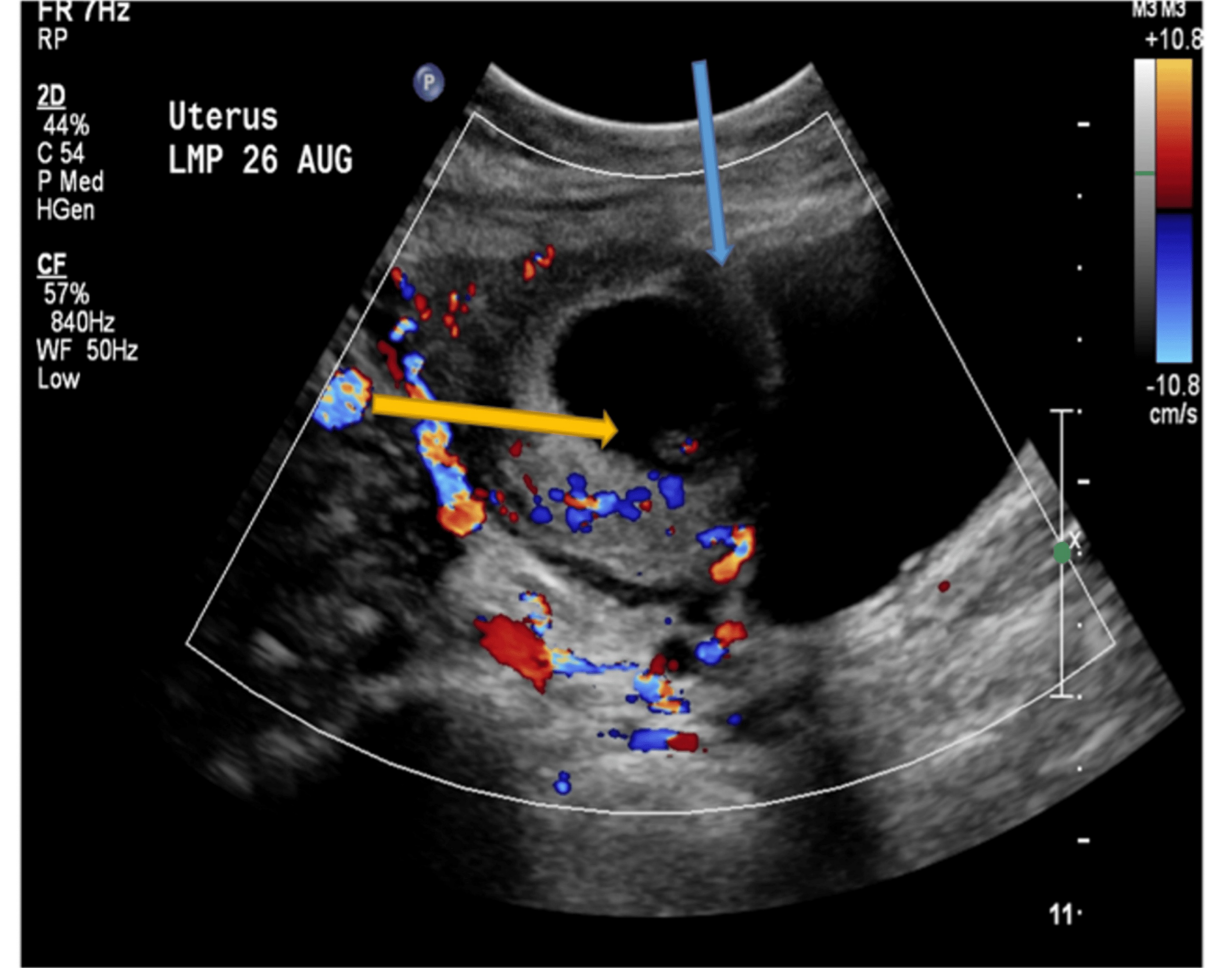
Axial transabdominal ultrasound demonstrating the characteristics of cesarean scar ectopic pregnancy Thin myometrium between gestational sac and urinary bladder (the blue arrow). The gestational sac contains a fetal pole with a positive heart color (the yellow arrow).

A thirty-six-year-old pregnant female, at a gestational age of seven weeks and one day, presented to the emergency department with a complaint of mild lower abdominal pain. The patient had a surgical history of five lower-segment cesarean sections and had one spontaneous first-trimester miscarriage; otherwise, there was an insignificant medical history. Beta-HCG was 83,468 mIU/mL. Transvaginal and transabdominal ultrasound (Figures 1, 2) showed marked thinning of the myometrium mantle in the anterior wall of the lower uterine segment. An oval gestational sac is close to the bladder wall with a viable fetus. Doppler revealed an irregularly shaped gestational sac embedded within the myometrium, accompanied by increased vascularity, high-velocity, and low-resistance blood flow patterns. Informed consent was obtained for suction evacuation under ultrasound guidance. The procedure was performed without any complications and a blood loss of 100 ml. Following the procedure, the patient was stable, with a complaint of minimal vaginal bleeding. The beta-HCG dropped to 25,339 mIU/mL (reference range 3,000 - 160,000 µ/L). She was discharged home with a regular follow-up in the clinic with serum beta-HCG till it reached an undetectable level one month after the procedure. The histopathology result showed immature chorionic villi and decidua consistent with products of conception. 

Case 2

**Figure 3:**
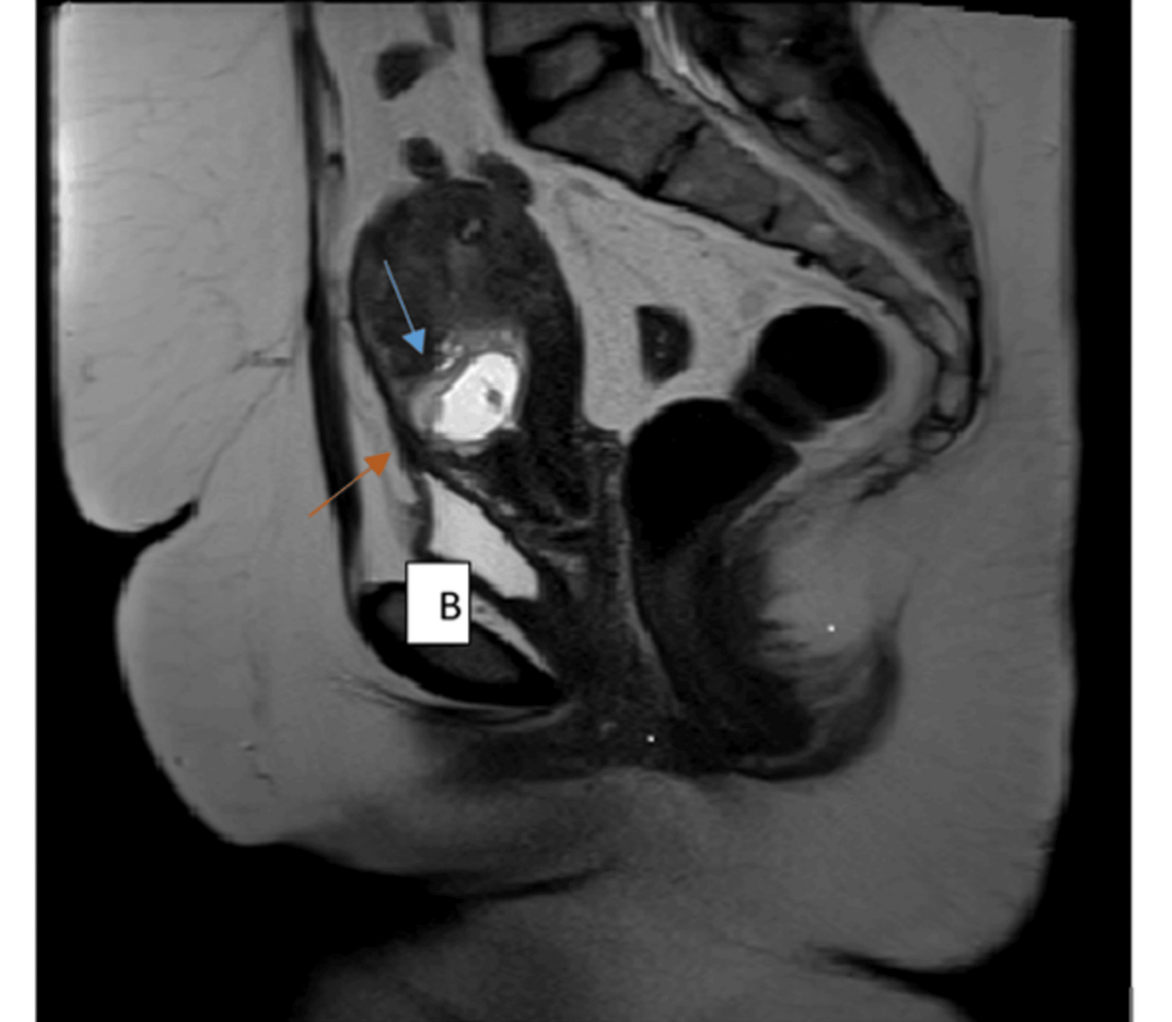
Coronal T2-weighted and axial haste T2W MRI of the pelvis for the same patient showing a partial septated uterus A sagittal T2-weighted MRI of the pelvis demonstrates a gestational sac (blue arrow) implanted within the anterior wall of the lower uterine segment in the region of the previous cesarean scar. Bladder wall integrity is preserved (B).

**Figure 4:**
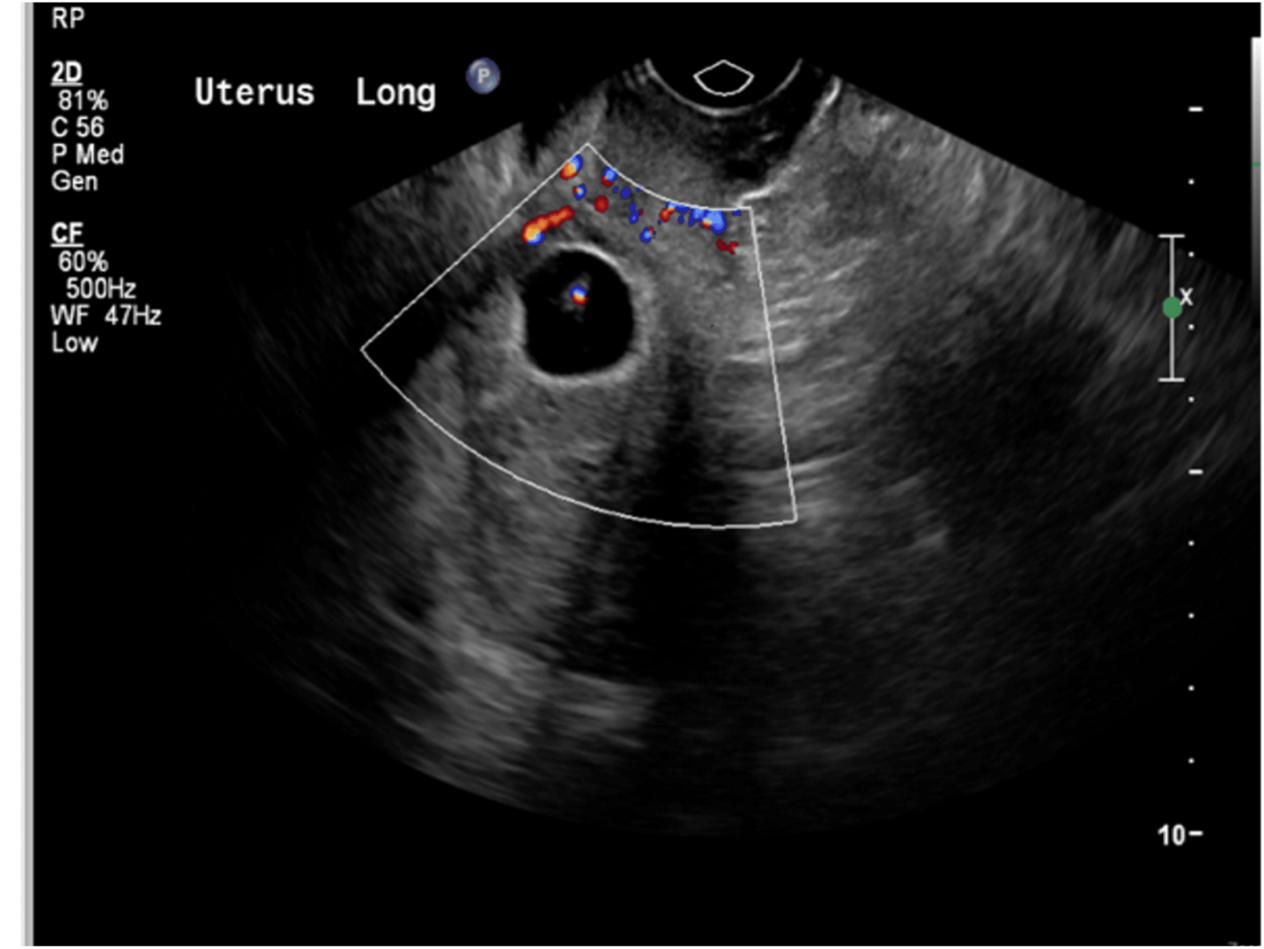
The uterine cavity and cervical canal were empty The anterior wall anterior to the gestational sac is thinned out, while the posterior wall is seen communicating with the endometrial cavity.

**Figure 5:**
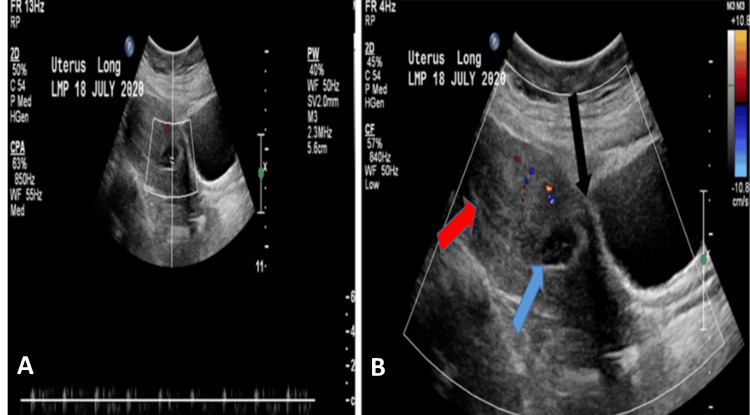
Longitudinal transabdominal ultrasound demonstrating the characteristic features of cesarean scar ectopic pregnancy Low-lying gestational sac (blue arrow) with positive cardiac activity, which is implanted in the area of the CS scar with thinning of the anterior myometrium between the gestational sac and uterine serosa (black arrow). The endometrium is empty (red arrow). A: characteristic features of CSEP; B: details about gestational sac, cardiac activity, and endometrium

A 34-year-old pregnant female, at a gestational age of seven weeks and four days, presented to the emergency department with a complaint of mild lower abdominal pain. The patient had a history of four previous lower-segment cesarean deliveries. The last one was complicated by bladder injury. The beta-HCG was 51,007 mIU/mL. The transvaginal ultrasound showed a bicornuate uterus; one of the cavities contained a gestational sac, while the other was empty (Figures 3, 4, 5). The gestational sac was implanted in the myometrium at the level of the previous cesarean scar; it showed a fetal pole with positive cardiac activity. An MRI of the pelvis showed a solitary ectopic gestational sac, likely to be implanted in the previous cesarean scar, with a fetal pole on a background of septated uterine anomaly. Informed consent for diagnostic hysteroscopy and suction evacuation of scar pregnancy was taken.

A diagnostic hysteroscopy was performed and localized the ectopic pregnancy at the scar area. A thick uterine septum and two uterine cavities with two opening ostia, one in each cavity, were seen. Suction evacuation of pregnancy under ultrasound guidance was performed without complications, with an estimated blood loss of 100 ml. The patient was stable the next day after surgery with minimal vaginal bleeding. The beta-HCG dropped to 30,121 mIU/mL (reference range 3,000 - 160,000 µ/L). She was discharged home with a follow-up in the clinic with serial beta-HCG till it reached 8 mIU/ml at 33 days following the procedure. The histopathology result showed immature chorionic villi and decidua consistent with products of conception. 

Case 3 

**Figure 6:**
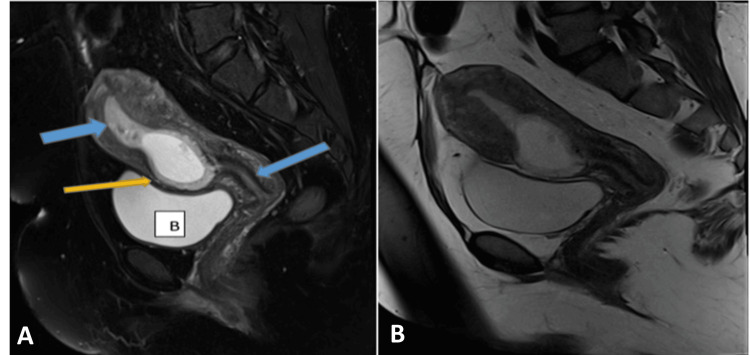
A sagittal T1 fat-suppressed imaging The sagittal T2-W demonstrating scar ectopic gestational sac with thinning of the anterior myometrium with low T2 signal (yellow arrow) and empty endometrial canal (blue arrows). A: ectopic gestational sac with thinning of the anterior myometrium marked with B; B: sagittal section

**Figure 7:**
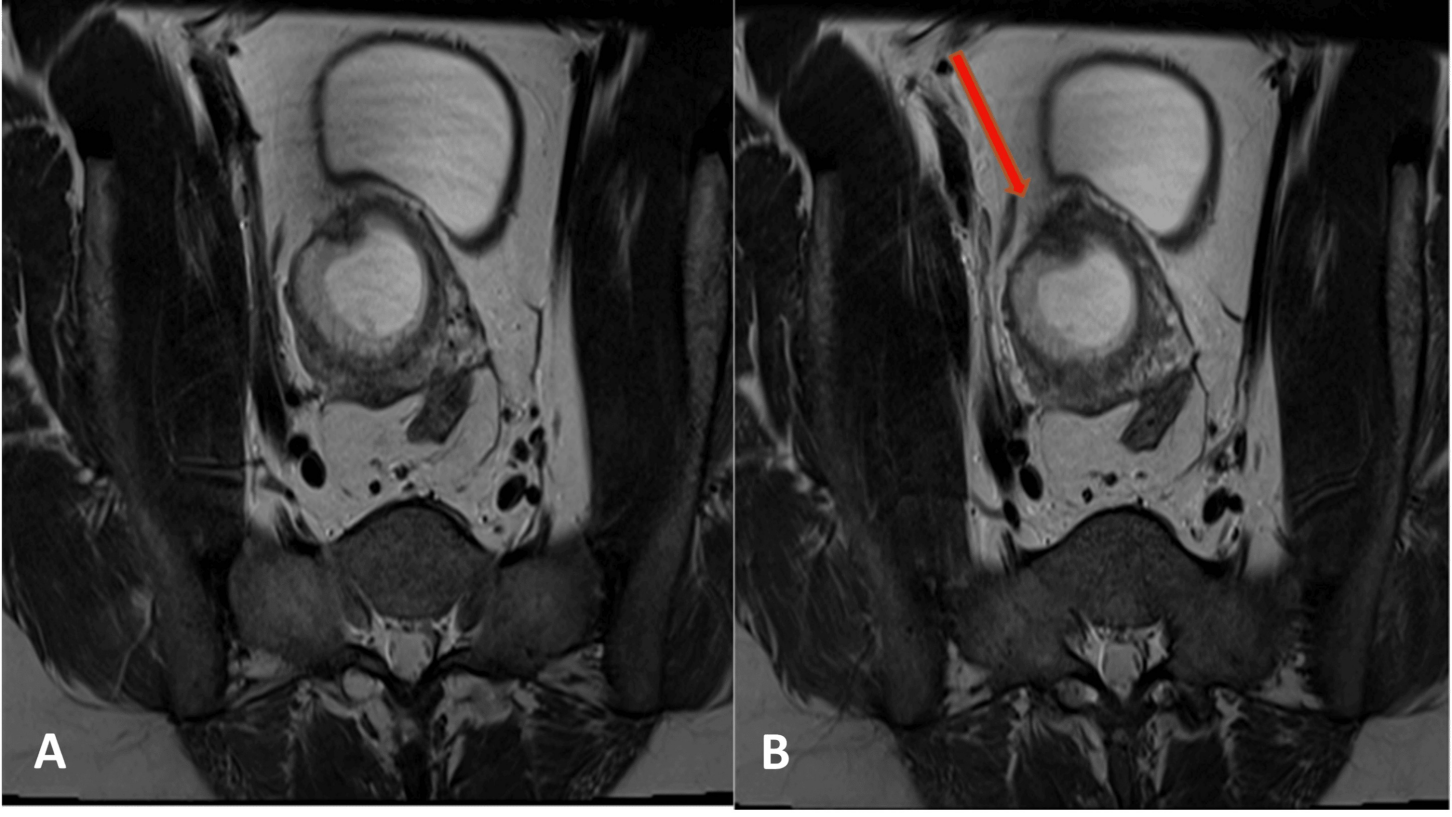
Axial T2-W demonstrating CS scar (red arrow) and thinning of the anterior myometrium with low T2 signal A: thinning of the anterior myometrium; B: cesarean scar (CS)

**Figure 8:**
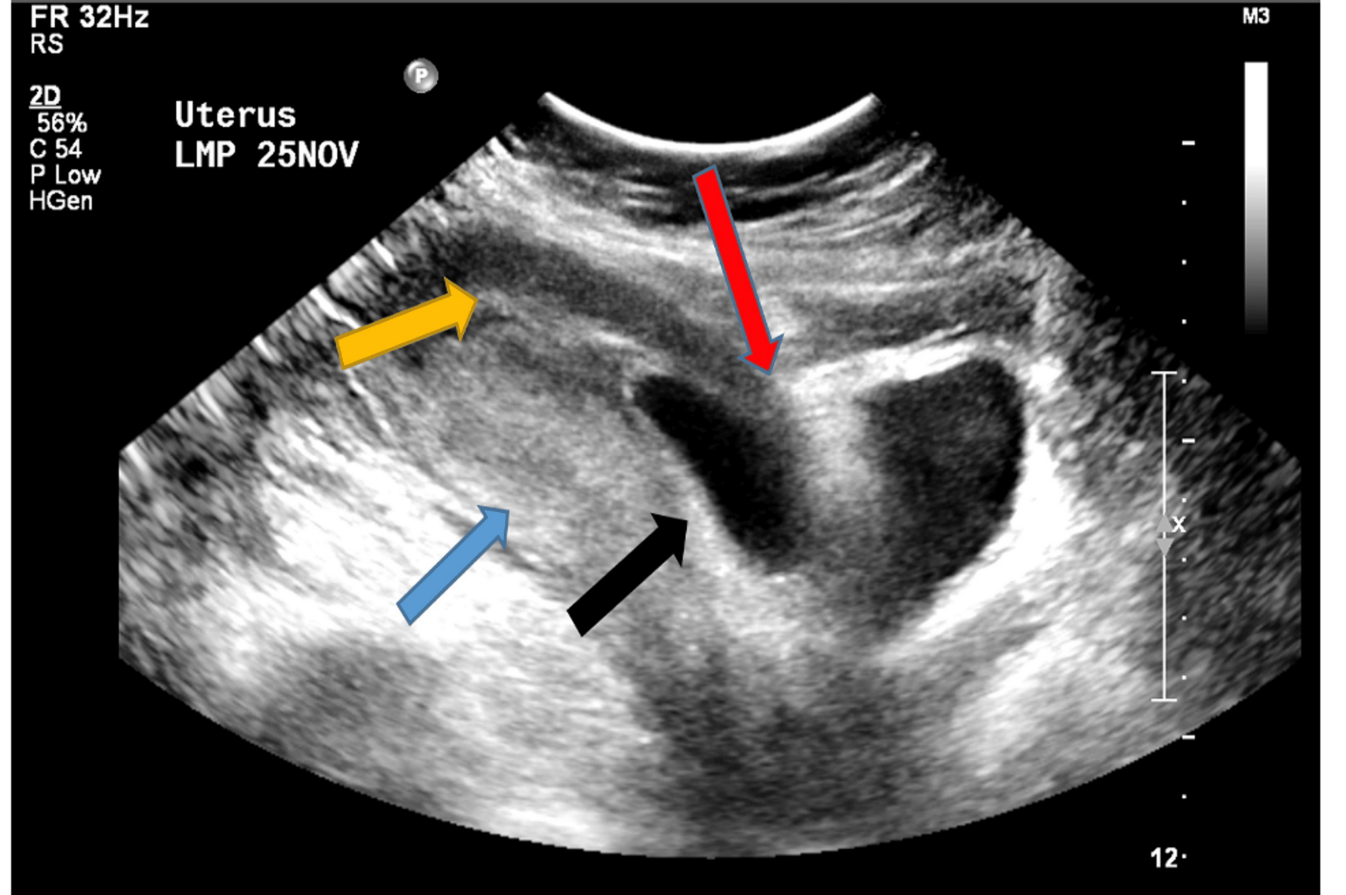
Sagittal transabdominal ultrasound showing an ectopic cesarean scar pregnancy The black arrow indicates a low-lying empty gestational sac with implantation in the area of the cesearean (CS) scar and thinning of the anterior aspect of the myometrium (red arrow). The yellow arrow indicates an empty endometrium. The blue arrow indicates the normal thickness of the posterior myometrial wall.

A 41-year-old pregnant woman, at a gestational age of six weeks and two days, was referred to our hospital with a suggestive diagnosis of cesarean scar ectopic pregnancy but has no complaint. The patient had a history of four cesarean deliveries and two dilatation and curettages but no significant medical history. The beta-HCG was 129,190 mIU/ml (reference range 200 - 32,000 µ/L). MRI was performed to confirm the diagnosis; it showed a low-lying gestational sac measuring 4.5 x 2.5 cm containing a small 1.1 cm fetal pole predominantly located within the anterior myometrium at the expected location of the cesarean scar with an almost imperceptible overlying myometrium (Figures 6, 7). A good proportion of the sac was oriented perpendicular to the long axis of the uterus. The transvaginal ultrasound showed a single intrauterine gestational sac with a fetal pole and positive cardiac activity. However, the gestational sac was noted to lie low at the level of the cesarean scar, and the anterior myometrium appears thin at this level (Figure 8).

Informed consent was secured for ultrasound-guided suction evacuation. The procedure was performed without complications. The estimated blood loss was 50 ml. On the first day after the procedure, the patient was stable with minimal vaginal spotting. The repeated serum beta-HCG dropped to 90,413 mIU/mL. She was discharged home with follow-up till beta-HCG was undetectable. The histopathology result showed immature chorionic villi and decidua consistent with products of conception. 

Case 4

A 43-year-old pregnant female, with a history of three previous cesarean sections and a current gestational age of seven weeks, was referred from another hospital with a suspected scar ectopic pregnancy and a complaint of brownish vaginal discharge. BHCG level was 27,721 mIU/mL (reference range 3,000 - 160,000 µ/L). Transvaginal ultrasound revealed a single sac was localized at the site of the cesarean scar. Informed consent was secured for suction evacuation under ultrasound guidance. The procedure was undertaken without complication. The estimated blood loss was 20 mL. The patient reported no pain or bleeding on the day after the procedure. Her BHCG levels dropped to 14,800 mIU/mL. She was discharged home with a plan for follow-up and beta-HCG monitoring. 

At a two-week follow-up at the clinic, the patient was asymptomatic. The beta-HCG levels further decreased to 18.7 mIU/mL. The histopathology result showed immature chorionic villi and decidua consistent with products of conception. The patient was reassured and discharged with advice for an early scan in the subsequent pregnancy. Four months later, the patient conceived again, and an early scan confirmed a normal intrauterine pregnancy. 

Case 5

A 40-year-old woman presented to the emergency department at eight weeks of gestational age with minimal vaginal bleeding. She had a surgical history of two previous cesarean sections. Transvaginal ultrasound confirmed the diagnosis of scar ectopic pregnancy, with a gestational sac containing a fetal pole measuring eight weeks and positive fetal heart activity. Her beta-HCG level was 86,811 mIU/mL. Informed consent was secured for suction evacuation under ultrasound guidance. The procedure was completed under ultrasound guidance without any complications. The estimated blood loss was 30 mL. On the first post-procedure day, the patient was asymptomatic. Beta-HCG levels had decreased to 29,591 mIU/mL (reference range 32,000 - 210,000 µ/L). Histopathology results from the products of conception revealed immature chorionic villi and decidua consistent with products of conception. The patient had regular follow-ups till beta-HCG was undetectable. 

Case 6

A 44-year-old woman with a history of five cesarean sections and one scar ectopic pregnancy was referred to our hospital as a case of scar ectopic pregnancy. She presented with brownish vaginal discharge. Beta-HCG was 7,232 (reference range 3,000 - 160,000 µ/L). Transabdominal and transvaginal ultrasound confirmed a cesarean scar ectopic pregnancy with a tiny fetal pole corresponding to seven weeks gestation and no fetal cardiac activity. Informed consent was secured for suction evacuation under ultrasound guidance.

The procedure was performed under general anesthesia with no complications. The estimated blood loss was 20 mL. Post-surgery, the patient remained asymptomatic. Beta-HCG dropped to 63 mIU/mL. She was discharged in stable condition. The beta-HCG at two weeks post-surgery was 2.1 mIU/mL. Histopathology results showed decidua and chorionic villi were consistent with the products of conception. 

Case 7

A 34-year-old woman, at nine weeks of gestational age, presented to the emergency department with a medical report suggesting a scar ectopic pregnancy. She had no complaints of abdominal pain or vaginal bleeding. The patient had a history of three cesarean deliveries and no significant medical history. The transvaginal ultrasound indicated an intrauterine gestational sac containing a viable fetus. The gestational sac was seen within the endometrium, low-lining close to the internal cervical OS. Subtle myometrial changes were seen at the lower portion of the uterus, mainly anterior to the lower part of the gestational sac. The crown-rump length (CRL) measuring 2.7 cm corresponds to nine weeks and three days with fetal heart rate detected. The beta-HCG was 185,466 mIU/mL (reference range 32,000 - 210,000 µ/L), and the hemoglobin level was 10.3 g/dL. Informed consent was obtained for suction evacuation under ultrasound guidance. During the procedure, there was excessive vaginal bleeding, which was controlled with balloon tamponade. Blood loss was estimated at 500 mL.

On the first postoperative day, the patient appeared well, with minimal vaginal bleeding and good urine output. The uterine balloon tamponade and the urine catheter were removed. Hemoglobin level was 6.4 g/dL. She received two units of blood transfusion, and hemoglobin increased to 9.1 g/dL. Beta-HCG dropped to 3,600 mIU/mL. She was discharged home with a plan for follow-up in the clinic with serum beta-HCG. Histopathology results showed decidua and chorionic villi were consistent with the products of conception. The patient had regular follow-ups till beta-HCG was undetectable. 

Case 8

A 44-year-old woman, seven weeks pregnant, presented to the emergency department, experiencing a complaint of minimal vaginal bleeding and mild lower abdominal pain. She had a history of four cesarean deliveries but an unremarkable medical history. The transvaginal ultrasound showed no endometrial gestational sac, and at the assumed region of the previous cesarean scar on the anterior wall of the lower uterine segment, a well-defined ectopic gestational sac showing a fetal pool within with no definite pulsations at the time of examination. The fetus's CRL revealed a gestational age of seven weeks and four days. The beta-HCG level was 9,355 mIU/mL (reference range 3,000 - 160,000 µ/L). Informed consent was obtained for suction evacuation under ultrasound guidance. Ultrasound-guided suction evacuation was successfully performed. Estimated blood loss was 50 mL. Post-operative, the patient remained asymptomatic with unremarkable examination findings. She was deemed fit for discharge with plans for follow-up with serum beta-HCG. At follow-up, on day ten post-procedure, the patient reported no pain or bleeding, and beta-HCG levels had dropped to 37.5 mIU/mL. Histopathology results confirmed retained products of conception consistent with the diagnosis. 

Case 9

A 32-year-old female with a history of two previous cesarean sections presented with lower abdominal pain. Her beta-hCG level was 126,993 mIU/mL. A transvaginal scan revealed a viable gestational sac located at the lower uterine segment at the cesarean scar site, with a crown-rump length (CRL) consistent with eight weeks and four days of gestation. The patient underwent ultrasound-guided suction evacuation without complications, experiencing minimal blood loss of 50 mL. Post-operatively, her beta-hCG levels dropped from 126,993 to 48,273 on day one and decreased to 21,443 on day 15 (reference range 32,000 - 210,000 µ/L). The histopathology report indicated features suggestive of a partial molar pregnancy. The patient is under ongoing follow-up with beta-hCG monitoring until the study publication date.

Case 10

A 38-year-old female with a history of two previous cesarean sections was 10 weeks pregnant and presented with brownish vaginal bleeding. Transvaginal ultrasound confirmed the diagnosis of cesarean scar ectopic pregnancy (CSEP), with a beta-hCG level of 15,329 mIU/mL (reference range 32,000 - 210,000 µ/L). She underwent ultrasound-guided suction evacuation, with a blood loss of 150 mL. Her beta-hCG level dropped to 5,520 post-operatively, and she was discharged in stable condition with serial follow-up, which showed complete resolution clinically and by beta-hCG. The histopathology report showed immature chorionic villi and decidua, indicating products of conception.

Key findings

All the patients treated with ultrasound-guided suction evacuation recorded a complete resolution of the ectopic pregnancies and no recurrence of symptoms or significant complications. Most of the patients experienced minimal blood loss during surgery, except in one case, which developed excessive blood loss, which was controlled by balloon tamponade.

Nine patients presented in this study showed rapid and sustained decline of beta-hCG determinants immediately after the operation, confirming the successful excision of the ectopic tissue. 

One case is still undergoing follow-up by beta-HCG till the date of study publication in which the histopathology confirmed partial molar pregnancy. 

All patients were satisfied and left the hospital in stable condition.

## Discussion

In this study, we presented a more straightforward and cost-effective method of treating cesarean scar ectopic pregnancy using ultrasound-guided suction evacuation. This approach has many benefits compared to the conventional operative approach, such as less healthcare cost, lesser recovery time, and higher patient satisfaction. 

This study revealed the successful CSEP management approach by using ultrasound-guided suction evacuation. The findings were comparable with many studies that have reported on the success and safety of different management options available for CSEP management [17-19]. Likewise, in a study by Timor-Tritsch et al., different approaches toward the excision of the CSEP, both laparoscopic and open for the excision of the ectopic gestational sac, were analyzed [20]. Their findings illustrated that patients who underwent laparoscopic surgery had a shorter duration of hospitalization and a faster return to normal activities than those who had open surgery. At the same time, the rates of successful pregnancies were similar for both interventions. However, each procedure still involved significant costs to the healthcare system and carried risks of adverse events.

On the other hand, our study illustrates the ultrasound-guided suction evacuation technique as effective in terminating CSEP, which is suggested as the first line of treatment. Our result equals the results of authors still getting their works published [21-23]. With more primary and repeat cesarean deliveries, the problem of cesarean scar ectopic pregnancies is also increasing [24].

Nonetheless, it is important to be truthful regarding our study limitations, such as a retrospective analysis of ten cases. Future studies with standardized protocols are needed to validate ultrasound-guided suction evacuation's relative safety and efficacy over conventional operative approaches.

Studies such as this are becoming more common due to the various attempted CSEP interventions. Advanced research is still required to determine the best course of action; however, ultrasound-guided suction evacuation is a reasonable and cost-effective method of dealing with this challenging clinical problem. 

Study limitations

The study acknowledges several limitations, including the small sample size and its retrospective nature, which may limit the generalizability of the findings to a larger population of women with cesarean scar ectopic pregnancy (CSEP). Additionally, while ultrasound-guided suction evacuation was the primary focus of this study, other treatment modalities for CSEP exist, such as medical management with methotrexate and surgical interventions like laparoscopic or open surgery. These alternatives were not explored in this study, which could provide a broader understanding of treatment efficacy and safety.

Patient choice upon counseling is another important factor that was not extensively covered in the study. Patients may have preferences for less invasive procedures or those with shorter recovery times, which could influence the choice of treatment modality. The study did not detail how patient preferences were integrated into the decision-making process, which could be a valuable area for future research.

The hospital policy regarding the management of CSEP, including the potential role of medical treatment or interventional radiology, was not explicitly discussed. Interventional radiology, such as uterine artery embolization, can be a valuable adjunct in managing CSEP, particularly in controlling hemorrhage. Future studies could benefit from exploring these aspects to provide a more comprehensive approach to CSEP management.

## Conclusions

Ultrasound-guided suction evacuation can be safely and effectively applied to CSEP management, and it remains one of the most cost-effective methods. Most clinical practitioners should be able to use this treatment option safely and achieve the desired results; hence, it appears to be a reasonable management option for most clinicians. Our review stresses the need to arrange first-trimester ultrasound examinations early for women with a history of cesarean section. This facilitates the quick determination of the gestational sac location, and intervention can be undertaken at the right time.

Based on this finding, women with a history of cesarean section must perform first-trimester ultrasound to look for the possibility of scar ectopic. And ensure that using the CSEP technique has successful results and minimal interventions, thus improving the quality of care. This study addresses a critical clinical need; as the prevalence of CSEP is bound to increase with the skyrocketing rates of cesarean deliveries, the incidence of this condition is bound to grow, and understanding better management strategies is essential.
